# Association of systemic bone mineral density and tibial cortical thickness with postoperative subsidence after surgical treatment of tibial plateau fractures

**DOI:** 10.1007/s00402-026-06528-5

**Published:** 2026-10-06

**Authors:** Mees K. Hesmerg, Joyce L. Benner, Pieter Joosse, Lucien C. M. Keijser

**Affiliations:** 1 Department of Orthopaedic Surgery, Amsterdam Movement Sciences, Musculoskeletal Health, Amsterdam University Medical Centers, Amsterdam, Netherlands; 2 Department of Orthopaedic Surgery, Noordwest Ziekenhuisgroep, Alkmaar, Netherlands; 3 Department of Trauma Surgery, Noordwest Ziekenhuisgroep, Alkmaar, Netherlands

**Keywords:** Tibial plateau fracture, Bone mineral density, Osteoporosis, Fracture subsidence, Tibial cortical thickness

## Abstract

**Introduction:**

Tibial plateau fractures are common fractures above the age of 50 years old and are frequently considered osteoporosis-associated fractures. However, the association between bone mineral density (BMD) and postoperative articular subsidence remains unclear. The primary objective was to assess whether there was an association between systemic BMD or tibial cortical thickness and articular subsidence after surgical treatment of tibial plateau fractures. Secondary objectives were to evaluate preoperative fracture displacement and the prevalence of reduced BMD in this cohort according to WHO criteria.

**Materials and methods:**

In this retrospective cohort study, patients treated surgically for low-energy tibial plateau fractures between 2017 and 2024 who underwent Dual-Energy X-ray Absorptiometry (DEXA) within 12 months before or after injury were included. DEXA-derived T-scores were used to assess systemic bone density and Tibial Cortical Thickness (TCT) was used as a measure of tibial cortical bone status. Fracture subsidence and preoperative displacement were measured in the coronal plane using articular depression. Multivariable linear regression analyses were performed adjusting for age, BMI and fracture comminution. The primary outcome was also adjusted for preoperative articular depression.

**Results:**

Fifty-nine patients were included in the analysis. There were 57 women and 2 men. Mean age was 63.9 years (SD ± 8.1) and mean BMI was 26.2 (SD ± 4.8). Schatzker type II was the most common fracture pattern (30/59, 50.8%), followed by Schatzker type VI (20/59, 33.9%). According to WHO criteria, 1.7% had normal bone mineral density, 49.2% had osteopenia and 49.2% had osteoporosis. There was no statistically significant association between postoperative subsidence and systemic BMD (*p* = 0.259) or TCT (*p* = 0.340). Nor was there a statistically significant association between preoperative articular depression and systemic BMD (*p* = 0.132) or TCT (*p* = 0.397).

**Conclusions:**

In this study, no statistically significant association was identified between systemic BMD or tibial cortical thickness and postoperative articular subsidence or preoperative depression, although the limited sample size warrants cautious interpretation. Osteoporosis and osteopenia were common in this selected cohort of DEXA-assessed patients above the age of 50 years with surgically treated low-energy tibial plateau fractures.

## Introduction

Tibial plateau fractures (TPFs) affect approximately 10–25 per 100,000 persons per year and pose a persistent challenge to treating physicians [1, 2]. Even after adequate reduction and fixation, rates of postoperative (secondary) articular subsidence are high and loss of reduction after open reduction and internal fixation (ORIF) is reported in 10–36% of cases [3–6], which may lead to unsatisfactory and unpredictable outcome.

Bone mineral density (BMD) is a key factor in bone strength and implant stability. Reduction of BMD naturally occurs in older individuals as a result of imbalance between bone resorption and bone formation, resulting in a gradual loss of bone mass and deterioration of bone microarchitecture [7, 8]. This decline is most prevalent in postmenopausal women and older men [8], and can be quantified using Dual-energy X-ray Absorptiometry (DEXA). Individuals with reduced BMD have a substantially increased lifetime risk of sustaining a fracture [9], and it has been previously suggested that low-energy TPFs in older individuals may represent osteoporotic fractures [3, 10, 11].

Given the high rates of postoperative articular subsidence and the known effects of reduced BMD on bone strength, it is plausible that BMD plays a role in the loss of reduction in TPFs. However, to date there are no reports on the association between postoperative articular subsidence of surgically treated TPFs and systemic BMD. Therefore, the primary aim was to investigate whether an association existed between systemic BMD or tibial cortical thickness and postoperative articular subsidence. Theoretically, reduced BMD may expose patients to greater articular depression after low-energy trauma; however, initial depression is also influenced by fracture morphology and the magnitude and direction of the forces involved. Therefore, a secondary aim was to explore the association between BMD and preoperative fracture displacement. A further secondary aim was to describe the frequency of osteopenia and osteoporosis in this cohort of surgically treated low-energy tibial plateau fractures.

## Methods

This was a retrospective cohort study of patients who were surgically treated for a tibial plateau fracture in a regional trauma facility in the Netherlands in the period from 2017 to 2024. This study was approved by the institutional review board (IRB) under local study ID L024-026 and informed consent was waived. This study was conducted according to the Declaration of Helsinki.

### Study population

The study included patients above the age of 50 years who underwent DEXA scanning 12 months prior to or after the tibial plateau fracture and who had a minimum of 100 days radiographic follow-up after surgery. According to Dutch national guidelines, all patients above the age of 50 who sustain a low-energy tibial plateau fracture receive an invitation to undergo a DEXA scan to assess BMD on a voluntary basis. Consequently, this study reflects a distribution of BMD for patients within this clinical pathway. If anti-osteoporotic treatment was already being administered at the time of the fracture, no invitation was sent and thus no patients on anti-osteoporotic treatment regimens were included. Trauma mechanisms were defined as low-energy when they did not meet the criteria for high-energy trauma according to Dutch national guidelines [12]. High-energy mechanisms included falls from height greater than 6 m, high-risk motor vehicle accidents, pedestrian or cyclist collisions at speed above 35 km/h, or motorcycle accidents at speed above 35 km/h.

### Patient and outcome variables

Electronic patient files were accessed and patient characteristics were extracted. Baseline characteristics included sex, age at time of fracture, BMI and DEXA results. DEXA results are expressed in a T-score, indicating how many standard deviations the BMD is below or above the average BMD of a healthy young adult of the same sex. According to the World Health Organization (WHO), T-scores are classified as normal (T-score > –1), osteopenia (T-score between −1 and −2.5), or osteoporosis (T-score ≤ –2.5) [13]. T-scores are measured at three anatomical sites: the lumbar spine and both hips. The lowest T-score determines the classification according to WHO reference standards and was used as a continuous variable in the analysis. For descriptive purposes, DEXA results were also classified according to the WHO reference standard into normal BMD, osteopenia and osteoporosis. Tibial Cortical Thickness (TCT) was used as a radiographic surrogate of tibial cortical bone status and measured by a single rater. TCT is calculated as the mean thickness of the medial and lateral cortex of the tibia measured at a distance of 10 centimeters distal to the articular surface, according to the previously described method of TCT measurement [14]. These measurements were taken on the initial anteroposterior posttraumatic radiograph of the affected tibia in all patients. All radiographs were obtained in the same institution using the same imaging protocol. No additional correction for rotation or magnification was applied. Computed Tomography (CT) imaging was used to assess fracture comminution. Comminution was defined as a fracture with three or more major fragments on CT imaging. CT imaging was available for all patients as this is part of routine care. Postoperative weight-bearing restrictions were determined by the treating surgeon based on fracture and fixation characteristics, with most patients following a 6-week non-weight-bearing protocol. Postoperative imaging was acquired on the day of surgery or the first day postoperatively. Postoperative articular subsidence was assessed using the follow-up radiograph closest to 12 months post-surgery.

Postoperative articular subsidence was defined as the difference between the direct postoperative radiograph and the follow-up radiograph. It was calculated as the signed change (Δ) in depression between the immediate postoperative and follow-up radiographs according to the method described by Assink et al. [15]. Intra-articular depression was measured perpendicular to the articular surface. In bicondylar fractures, the highest preserved portion of the articular surface was used to establish this reference line. Pre- and postoperative measurements of depression were performed by a single rater (MH, orthopaedic resident). To assess measurement reliability, a second rater (LK, consultant orthopaedic surgeon) assessed a sample of 15 consecutive patients. Extraction of patient and clinical characteristics from the electronic patient records, including DEXA results, and radiographic measurement of subsidence was performed in separate assessment sessions, reducing the likelihood of direct observer bias. The second rater was blinded to the measurements of the first rater.

### Statistical analysis

Descriptive statistics were used to assess baseline characteristics; continuous values are given as mean (SD) or median [IQR] and integer characteristics as n (%). Inter-rater reliability for depression was assessed using intraclass correlation coefficients (ICCs) with 95% confidence intervals (95% CI) using a two-way random effects model for absolute agreement. For all statistical tests, an alpha value of *p* < 0.05 was used. Statistical analysis was performed using IBM SPSS Statistics for Windows Version 28.0 (IBM Corp., N.Y., USA).

The associations between T-scores from DEXA scans, TCT, and postoperative articular subsidence, measured in millimeters as Δ depression, were first assessed using a univariable regression analysis. Next, multivariable linear regression analyses, adjusting for age, BMI, and comminution were used. As a secondary outcome, associations between T-scores, TCT, and preoperative depression was studied using the same method. The model for postoperative articular subsidence was also adjusted for preoperative depression. These covariates were selected a priori as potential confounders based on clinical relevance and previous literature [3–17]. Sex was initially considered as a potential confounder, but was not included in the final multivariable models because only two patients were male. As a sensitivity analysis, the primary multivariable models were additionally adjusted for the interval between surgery and the follow-up radiograph used to assess postoperative subsidence. In addition, postoperative subsidence > 2 mm as a binary outcome was analyzed as a sensitivity analysis using univariable logistic regression because of the limited number of events. Lastly, a sensitivity analysis was performed which adjusted the primary model for the time between the fracture and DEXA assessment. Results were considered consistent when the direction of the effect estimate and the overall interpretation were comparable to the main analyses. Continuous variables were checked for normality. Model assumptions, including linearity and homoscedasticity, were evaluated via visual inspection of residual plots and Q-Q plots.

## Results

In the period from 2017 to 2024, there were 159 patients above the age of 50 years surgically treated for a tibial plateau fracture. For 85 patients, DEXA imaging was available. Of these, eight patients had insufficient radiographic follow-up and 18 patients underwent DEXA assessment more than one year before or after the fracture. This resulted in 59 patients with a tibial plateau fracture who were included in the analysis. There were 57 women and two men. Mean age was 63.9 ± 8.1 years and mean follow-up was 310 ± 134 days (range 101–858). The most common fracture patterns were Schatzker type II (50.8%) and type VI (33.9%). Complete patient characteristics are given in Table 1. Among the included patients, 58 of 59 had reduced BMD according to WHO criteria; 49.2% had osteoporosis and 49.2% had osteopenia. Three patients underwent DEXA assessment prior to the fracture (364, 321 and 272 days before), and 56 after the fracture (range 29 to 364 days). Postoperatively, 53 patients followed a non-weight-bearing protocol, 5 were allowed 25% weight-bearing, and 1 patient was allowed 50% weight-bearing.

**Table 1:** Patient characteristics (*n* = 59)

Sex	
Male	2 (3.4)
Female	57 (96.6)
Age, years*	63.9 ± 8.1
BMI, kg/m^2^*	26.2 ± 4.8
Chronic kidney disease	2 (3.4)
Diabetes Mellitus	
Type I	0 (0)
Type II	6 (10.2)
Active smoker	10 (16.9)
Cardiovascular disease	16 (27.1)
Follow up time, days*	310 ± 134
Time to DEXA**	128 [88–203]
T-score on DEXA*	–2.5 ± 0.8
Tibial Cortical Thickness, mm*	2.9 ± 0.88
Type of fixation	
Locking plate	49 (83.1)
Screws	10 (16.9)
WHO BMD criteria	
Normal	1 (1.7)
Osteopenia	29 (49.2)
Osteoporosis	29 (49.2)
Comminuted fracture	27 (45.8)
Schatzker type	
Type I	1 (1.7)
Type II	30 (50.8)
Type III	1 (1.7)
Type IV	3 (5.1)
Type V	4 (6.8)
Type VI	20 (33.9)
Pre-operative depression (mm)**	6 [3–14]

*Given in mean ± SD

**Given in median [Q1–Q3]

**Fig. 1:**
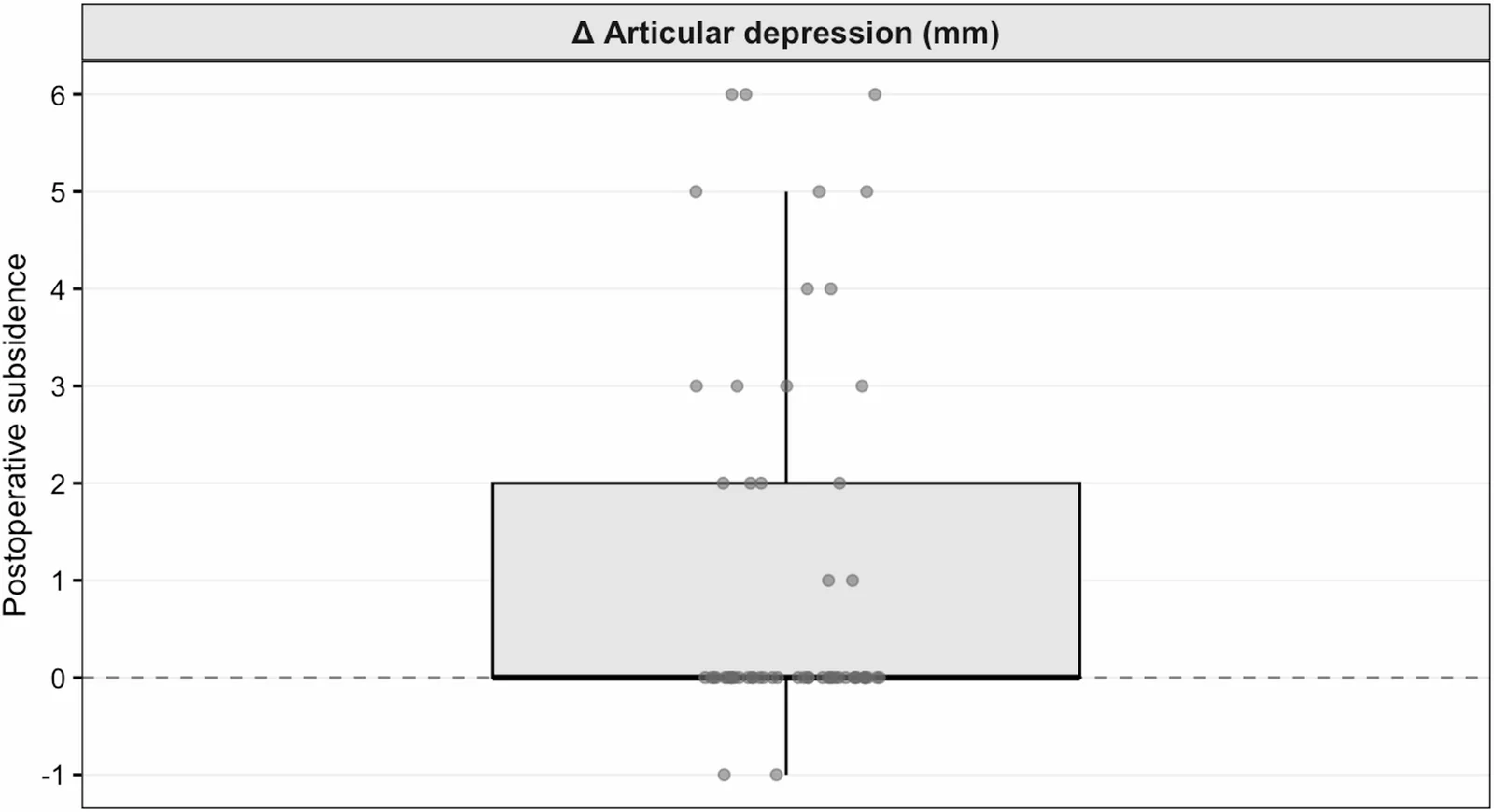
Distribution of postoperative articular subsidence (*n* = 59) expressed as signed change (Δ) in articular depression between immediate postoperative and follow-up radiographs. Negative values indicate apparent elevation of the articular surface

The median signed change in articular depression was 0 mm (IQR 0.0–2.0) (Fig. 1). In total, 18 patients suffered from postoperative articular subsidence (30.5%) of which 12 had subsidence >2 mm (20.3%) (Fig. 1). The inter-observer reliability analysis for articular depression yielded an ICC of 0.774 (95% CI 0.307–0.925). Both the univariable and the multivariable analyses revealed no statistically significant associations between DEXA-derived T-score and postoperative articular subsidence (β 0.340, 95% CI – 0.26–0.94, *p* = 0.259) or TCT and postoperative articular subsidence (β 0.258, 95% CI – 0.28–0.80, *p* = 0.340) (Table 2). Sensitivity analyses using postoperative articular subsidence > 2 mm as a binary outcome showed no statistically significant association between DEXA-derived T-score and postoperative articular subsidence > 2 mm (OR 1.04, 95% CI 0.482–2.231, *p* = 0.926) or TCT and postoperative articular subsidence > 2 mm (OR 1.31, 95% CI 0.624–2.749, *p* = 0.476). The sensitivity analysis that adjusted the model for the time between surgery and the follow-up radiograph did not substantially alter the associations between DEXA-derived T-score and postoperative articular subsidence (β 0.339, 95% CI – 0.265–0.943, *p* = 0.265) or between TCT and postoperative articular subsidence (β 0.283, 95% CI – 0.271–0.836, *p* = 0.310). Adjustment for the interval between fracture and DEXA assessment also did not alter the associations between DEXA-derived T-score and postoperative subsidence (β 0.345, 95% CI -0.288 to 0.978, *p* = 0.279). All sensitivity analyses were consistent with the primary continuous outcome analysis. There was also no statistically significant association between systemic BMD or TCT and preoperative depression (Table 3). Visual inspection of the residual plots did not indicate major violations of linearity, homoscedasticity, or residual normality.

**Table 2:** Uni- and multi-variable linear regression for subsidence

	Univariable			Multivariable		
	*β*	95% CI	*p*	*β*	95% CI	*p*
***A: Systemic bone density (DEXA)***						
T-score on DEXA	0.014	– 0.59; 0.62	0.962	0.340	– 0.26; 0.94	0.259
*Preoperative depression (mm)*	–	–	–	*0.133*	*0.08; 0.19*	*< 0.001*
*Age (years)*	–	–	–	*0.005*	*– 0.06; 0.07*	*0.865*
*BMI (kg/m* ^*2*^ *)*	–	–	–	*– 0.055*	*– 0.15; 0.04*	*0.271*
*Comminution (ref: no)*	–	–	–	*0.091*	*– 1.01; 0.82*	*0.843*
***B: Tibial Cortical thickness***						
TCT (mm)	0.376	– 0.19; 0.94	0.189	0.258	– 0.28; 0.80	0.340
*Preoperative depression (mm)*	–	–	–	*0.124*	*0.07; 0.18*	*< 0.001*
*Age (years)*	–	–	–	*0.002*	*– 0.06; 0.06*	*0.960*
*BMI (kg/m* ^*2*^ *)*	–	–	–	*– 0.045*	*– 0.14; 0.05*	*0.351*
*Comminution (ref: no)*	–	–	–	*– 0.178*	*– 1.11; 0.76*	*0.704*

**Table 3:** Uni- and multi-variable linear regression for preoperative articular depression

	Univariable			Multivariable		
	*β*	95% CI	*p*	*β*	95% CI	*p*
***A: Systemic bone density (DEXA)***						
T-score on DEXA	– 1.596	– 4.41; 0.95	0.214	– 2.182	– 5.04; 0.68	0.132
*Age (years)*	–	–	–	*– 0.135*	*– 0.43; 0.16*	*0.363*
*BMI*	–	–	–	*0.114*	*– 0.37; 0.60*	*0.638*
*Comminution (ref: no)*	–	–	–	*3.103*	*– 1.17; 7.42*	*0.150*
***B: Tibial Cortical thickness***						
TCT (mm)	1.487	– 0.94; 3.91	0.225	1.123	– 1.51; 3.76	0.397
*Age (years)*	–	–	–	*– 0.034*	*– 0.33; 0.26*	*0.816*
*BMI*	–	–	–	*– 0.004*	*– 0.48; 0.47*	*0.986*
*Comminution (ref: no)*	–	–	–	*2.915*	*– 1.53; 7.36*	*0.194*

## Discussion

The primary aim of this study was to investigate whether there was an association between postoperative articular subsidence and systemic BMD measured on DEXA or tibial cortical thickness. No statistically significant association between BMD or TCT and postoperative articular subsidence of surgically treated tibial plateau fractures was identified in this cohort of patients above the age of 50 who underwent DEXA assessment shortly prior to or after the fracture. Similarly, BMD or TCT and preoperative displacement showed no statistically significant association. Most patients who were included in the study had reduced BMD. To our knowledge, this study is the first to investigate the association between DEXA-derived T-scores or TCT and articular subsidence in surgically treated TPFs.

The lack of a statistically significant association between BMD and postoperative articular subsidence challenges the conceptual assumption that low systemic bone density compromises subchondral support in tibial plateau fractures [6]. Yet, the confidence intervals were wide and therefore do not exclude potentially clinically relevant associations. Possibly, modern fixation strategies may compensate for the mechanical consequences of reduced bone strength, although this hypothesis was not directly evaluated in this study.

An important consideration in the interpretation of the results is that DEXA-derived T-scores reflect systemic bone mineral density rather than local metaphyseal or subchondral bone quality at the fracture site. Therefore, we also incorporated measurements of tibial cortical thickness, which has been previously shown to correlate with systemic BMD [14, 18]. Previous work by Gausden et al. [16] used locally measured Hounsfield Units on CT imaging to assess BMD. Their findings also suggested that subsidence was not more prevalent in reduced BMD. Furthermore, they also found no difference in patient-reported outcome measures (PROMs). Although both this study and the study by Gausden et al. are retrospective reports, the results indicate that patients with reduced BMD do not necessarily have an increased risk of inferior outcome than patients with normal BMD. Neither systemic BMD or TCT showed a statistically significant association with secondary subsidence.

In the present sample, nearly all patients who were included had osteoporosis or osteopenia (98.3%). This study used a convenience sample in which DEXA imaging was based on voluntary patient response, which introduces a risk of selection bias. Yet, this result suggests that rates of reduced BMD could be high in patients above the age of 50 years who suffer a tibial plateau fracture after a low-energy trauma. This finding raises questions about the classification of low-energy tibial plateau fractures in older individuals. Currently, such fractures are not typically addressed as fragility fractures by organizations such as the National Institute for Health and Care Excellence (NICE) and International Osteoporosis Foundation [19, 20]. The exceptionally high rate of reduced BMD in this study could be hypothesis-generating, suggesting that low-energy tibial plateau fractures in the elderly may be an indicator of reduced BMD. However, since only 59 out of a total of 203 patients within the study period could be included in our study, the results should be interpreted within this cohort, rather than population-based estimates. Since this is the first study to assess the frequency of reduced systemic BMD in low-energy TPF patients above the age of 50, this finding requires confirmation through future studies.

No statistically significant association between reduced BMD and increased preoperative displacement was identified. Weaker metaphyseal bone may be less capable of handling the forces that occur in trauma, resulting in greater deformity or displacement even in low-energy trauma mechanisms. These findings highlight the complexity of fracture mechanics in osteoporotic bone and underscore the importance of comprehensive multivariate modeling when assessing the role of bone density.

A limitation of this study is the use of a retrospective design using a convenience sample. No a priori sample size calculation was performed because the study included all patients available during the study period. Patients undergo DEXA imaging on a voluntary basis; therefore, not all patients with a tibial plateau fracture will undergo BMD assessment. Possibly, patients with a higher suspicion of reduced BMD were more likely to undergo DEXA imaging. This may have caused overrepresentation of reduced BMD in our sample. Therefore, these proportions should be interpreted as descriptive findings within our sample, rather than population-based estimates. The sample size limited statistical precision and the ability to detect smaller associations. The nonsignificant findings had wide confidence intervals, which cannot exclude the presence of an association. Medication documentation was not consistently available for a small proportion of the source population, which prevented reliable assessment of the number of patients who did not receive an invitation for DEXA assessment because of anti-osteoporotic treatment. Secondly, the timing of DEXA scans up to 12 months prior to or after the fracture may not perfectly reflect bone density at the time of injury. Immobilization or fracture-related bone loss could have influenced post-fracture measurements, although the sensitivity analysis was consistent with the primary model. The cohort was also predominantly female, which limits the generalizability to male populations. Postoperative articular depression and subsidence were measured on plain radiographs. Two-dimensional imaging may underestimate articular incongruity and is sensitive to differences in radiographic projection. Differences in positioning and radiographic projection between postoperative and follow-up imaging may have contributed to measurement variability. Furthermore, TCT was measured by a single rater and ICC was not assessed, which may have introduced variability. The inter-observer variability analysis for articular subsidence was based on only 15 patients, this resulted in a wide confidence interval with imprecise estimates of the true reliability, intra-rater reliability was not assessed. Additionally, radiographic measurements of TCT may be influenced by projection and magnification. Although TCT has been associated with systemic BMD and osteoporosis, it has not been validated as a measure of subchondral bone quality in tibial plateau fractures. Lastly, residual confounding is possible because several mechanical and treatment-related determinants of subsidence such as reduction quality, fixation methods, specific fracture morphology, or weight-bearing protocol could not be included in the model. Future research should focus on prospective studies of consecutive patients to reduce selection bias and improve generalizability.

## Conclusion

In this study, no statistically significant association was identified between systemic BMD or tibial cortical thickness and postoperative articular subsidence or preoperative depression, although the limited sample size warrants cautious interpretation. Osteoporosis and osteopenia were common in this selected cohort of DEXA-assessed patients above the age of 50 years with surgically treated low-energy tibial plateau fractures. These findings do not support the use of BMD or TCT alone for estimating the risk of postoperative subsidence.

## Data Availability

No datasets were generated or analysed during the current study.
